# Tailored Synthesis of Core-Shell Mesoporous Silica Particles—Optimization of Dye Sorption Properties

**DOI:** 10.3390/nano8040230

**Published:** 2018-04-10

**Authors:** Andrzej Baliś, Szczepan Zapotoczny

**Affiliations:** Faculty of Chemistry, Jagiellonian University, Gronostajowa 2, 30-387 Krakow, Poland; balis@chemia.uj.edu.pl

**Keywords:** core-shell nanoparticles, mesoporous silica, surfactant templating synthesis, dye adsorption, radially ordered mesopores

## Abstract

Monodisperse spherical silica particles, with solid cores and mesoporous shells (SCMS), were synthesized at various temperatures using a one-pot method utilizing a cationic surfactant template. The temperature of the synthesis was found to significantly affect the diameters of both the cores (ca. 170–800 nm) and shells (ca. 11–80 nm) of the particles, which can be tailored for specific applications that require a high specific surface area of the nanocarriers (mesoporous shells) and simultaneously their mechanical robustness for, e.g., facile isolation from suspensions (dense cores). The applied method enabled the formation of the relatively thick mesoporous shells at conditions below room temperature. Radially ordered pores with narrow distributions of their sizes in 3–4 nm range were found in the shells. The adsorption ability of the SCMS particles was studied using rhodamine 6G as a model dye. Decolorization of the dye solution in the presence of the SCMS particles was correlated with their structure and specific surface area and reached its maximum for the particles synthesized at 15 °C. The presented strategy may be applied for the fine-tuning of the structure of SCMS particles and the enhancement of their adsorption capabilities.

## 1. Introduction

Mesoporous silica nanoparticles (MSNs), especially with ordered mesopores, have attracted increasing interest as carriers in, e.g., biomedical, catalytic, and optical applications, due to their desired properties, such as their high specific surface area, well-defined and tunable pore-sizes, transparency, or biocompatibility [1,2,3,4,5,6]. MSNs obtained for the first time in the early 2000s [7,8,9,10] have been developed towards various dimensions, morphologies and pores sizes. MSNs are commonly synthesized using a sol–gel reaction of silica species in a surfactant templating method [11]. The Stöber method [12], involving the hydrolysis of tetraalkyl silicates in a mixture of alcohol and water using ammonia as a catalyst, was also developed for the synthesis of submicrometer mesoporous silica particles originally by Grun et al. [13]. MSNs with radially aligned mesopores were also later synthesized using a modified Stöber approach [14]. Such ordered mesopores may be formed thanks to the self-organization of the micellar rods into a hexagonal matrix at proper conditions, which serves as a template for the organization of the silica precursors.

Among the various modifications of MSNs, the structures containing solid cores and mesoporous shells (SCMS) are especially useful for applications requiring recycling of the nanoparticles, serving as carriers or dispersible reactors [15] as well as for sorption applications [16]. Such structures provide higher density and mechanical durability [17] necessary for, e.g., centrifugation or filtration purposes, but also enable the formation of hollow mesoporous particles after selective removal of the cores [18]. 

The fabrication of SCMS silica particles of the desired properties requires careful optimization of the synthetic conditions, which include variation of composition, pH of the reaction mixture, temperature, or solvent selection. While pH was found to be the most influential factor determining the diameters of MSNs [19,20], temperature was also used to regulate the size of MSNs [3], although the trends may differ, likely due to the different mechanisms of their synthesis [1,21]. Nevertheless, the synthesis of SCMS silica particles has been mainly realized at room or higher temperatures and the influence of lower temperature on the size and properties of the mesoporous shell has been often neglected [22,23,24]. However, the temperature of the synthesis and concentration of the applied surfactant should significantly influence the properties of the mesoporous phase in the SCMS particles, obtained using micellar template methods, because critical micellar concentration (CMC), Krafft point, as well as the stability of the micelles that depend on those parameters [25,26]. While decreasing the temperature might improve the ordering of micellar templates, the Krafft temperature (T_K_) limits the applicability of a given surfactant at lower temperatures (solubility of a surfactant is lower than CMC below T_K_). This is particularly important for ionic surfactants with long aliphatic chains. 

Herein, we present a synthesis of monodisperse SCMS silica submicrometer particles at various temperatures enabling tuning of their sizes and properties. The syntheses at conditions below room temperature have been especially neglected in the literature and here they have been shown to result in particles of improved sorption properties. Model dye molecules were adsorbed in order to test the sorption abilities of the synthesized particles, which can be later used in wastewater treatment or as dispersible photoreactors. The observed correlations were explained mainly in terms of variations of the behavior of surfactant templates that influence, e.g., shell thickness, specific surface area, and the pore sizes of the synthesized SCMS particles.

## 2. Materials and Methods

### 2.1. Materials

Tetraethoxysilane (TEOS, 98%, GC), hexadecyltrimethylammonium bromide (CTAB, 98%), and poly(ethyleneimine) (PEI, branched, M_n_ ≈ 10,000 g/mol) were purchased from Sigma Aldrich (St. Louis, MS, USA). Rhodamine 6G (Rh6G, 99%) was purchased from Acros Organics (Geel, Belgium). Ammonia solution (30%, p.a.) and ethanol (96%, p.a.) were purchased from Chempur (Piekary Slaskie, Poland). Deionized water was used in all procedures.

### 2.2. Apparatus

N_2_ sorption studies were conducted at −196 °C using a 3Flex v1.00 (Micromeritics, Norcross, GA, USA) automated gas adsorption system. Prior to the analyses, the samples were degassed under vacuum at 350 °C for 24 h. Fourier-Transform Infrared Spectroscopy (FT-IR) spectra were recorded using a Nicolet iS10 FT-IR spectrometer (Thermo Fisher Scientific, Waltham, MA USA) with an ATR accessory. UV-VIS spectra were recorded on a Varian Cary 50 (Palo Alto, CA, USA) UV-VIS spectrophotometer. Transmission Electron Microscopy (TEM) measurements were carried out by means of a FEI (Lausanne, Switzerland) Tecnai Osiris microscope with an X-FEG Schottky field emitter operated at 200 kV. Scanning Transmission Electron Microscopy (STEM) imaging was performed using a high-angle annular dark-field (HAADF) detector. Before analysis, the samples were ultrasonically dispersed in ethanol and dropped on a Lacey type copper grid (200 mesh) (Agar Scientific, Stansted, UK). Conductivity measurements were performed using a multifunction computer meter CX-741 (Elmetron, Zabrze, Poland). A 0.01 M KCl solution of known specific conductivity was used to determine the specific conductivities of the measured solutions. Centrifugation was carried out with an MPW-250 (MPW Med. Instruments, Warsaw, Poland). A Spin 150 wafer spinner (APT GmbH, Korbach, Germany) was used for spin-coating.

### 2.3. Procedures

#### 2.3.1. Synthesis of SCMS Particles

The synthesis of solid core mesoporous shell (SCMS) silica particles was based on the procedure reported in Reference [24], which was then modified here. In the first step, 100 mL of ethanol, 8 mL of water, and 4 mL of ammonia solution (catalyst) were mixed and kept at a given temperature (8 °C, 15 °C, 22 °C, 45 °C, or 60 °C) in covered beakers. Subsequently, 6 mL of TEOS was added to the mixture that was stirred for 6 h at the same temperature leading to the formation of the suspension of particles (solid cores). A portion of the obtained suspension was centrifuged (5 min, 7900 RCF) and the obtained particles were washed with ethanol and dried at 50 °C overnight. The resulting particles were named, indicating the temperature of the synthesis (e.g., 8 °C-SC). One-hundred milliliters of the suspension prepared in the first step was diluted with 200 mL of water and 30 mL of CTAB surfactant solution in ethanol/H_2_O (1:2 *v*/*v*) was added. The total concentration of CTAB in the reaction mixture was equal to 0.01 M. After 30 min of vigorous stirring, 2.15 mL of TEOS was added and the mixture was stirred overnight at a given temperature (the same as for the formation of cores). The resulting core-shell particles were isolated and purified the same way as the core particles and named to indicate the temperature of the formation (e.g., 8 °C-SCMS). To remove the surfactant template SCMS particles were heated in air at 550 °C for 4 h (heating rate: 2 K/min), resulting in the formation of the calcined samples, SCMS-C. Additionally, for the samples formed at 22 °C, four fractions were collected after 6, 12, 24, and 48 h of mesoporous shell formation.

#### 2.3.2. Rhodamine 6G Adsorption Studies

For the adsorption studies, 7.5 ± 0.05 mg of a given SCMS-C sample was added to 8 mL of Rh6G aqueous solution (c = 5 mg/L, absorbance at 529 nm, A = 0.84) and sonicated for 15 min, and then shaken for 1 h using a multi-vortex v32. Afterwards, the suspension was centrifuged (5 min, 7900 RCF) and the UV-VIS spectrum of the supernatant was measured. 

#### 2.3.3. Scanning Electron Microscopy Measurements

Scanning electron microscopy studies were performed using a Phenom Pro microscope (Phenom World, Eindhoven, The Netherlands) working at an operational voltage of 10 kV. Silicon substrate was used for the deposition of the obtained particles. The wafer was purified by immersing it in the “piranha” solution (H_2_O_2_/H_2_SO_4_ 1:3 *v*/*v*) for 15 min. This procedure must be carried out with caution, as it is a highly corrosive and oxidative mixture. The wafer was subsequently washed with water and left for 5 min in a PEI aqueous solution (2 g/L) to reverse the surface charge. Afterwards, the coated substrate was washed again with water and the respective suspension of particles was deposited on it by spin-coating (2000 RPM, 1 min). For high-resolution imaging, such prepared dried samples were coated with a nanometric layer of gold. Images of at least 100 particles for each sample with circularity higher than 0.9 were captured at a magnification equal to 40,000× or larger. Diameters of the particles were determined based on the surface areas of the particles from the SEM images using automatic detection offered by Fiji, a Java-based image-processing program developed at the National Institutes of Health.

## 3. Results and Discussion

### 3.1. Synthesis and Microscopic Characterization of SCMS Particles

**Figure 1:**
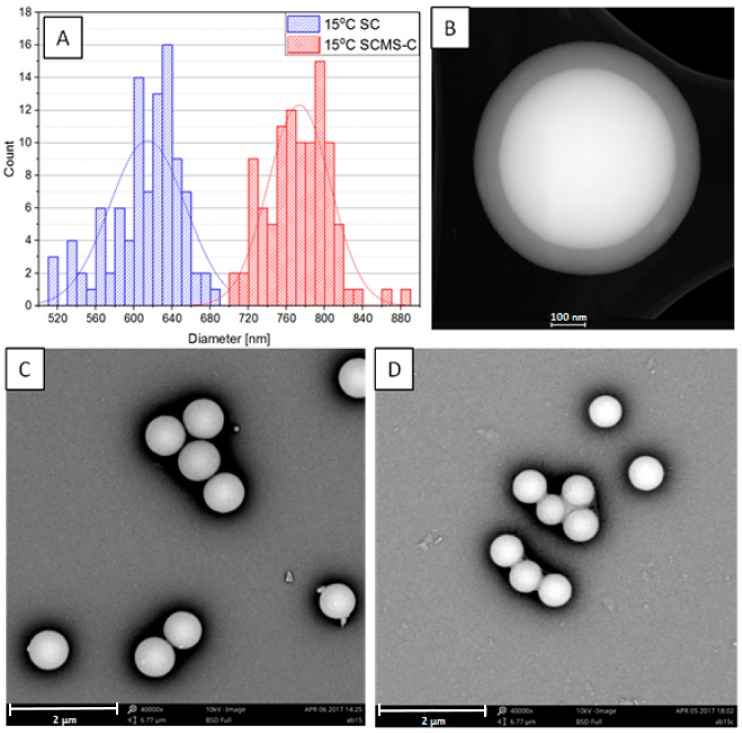
(**A**) Distributions of the diameters of 15 °C-SCMS-C and 15 °C-SC particles as determined from their respective SEM images; (**B**) STEM HAADF image of 15 °C-SCMS-C; (**C**) SEM image of 15 °C-SCMS-C; and (**D**) SEM image of 15 °C-SC.

The non-porous silica particles (solid cores) obtained in the first step of the synthesis were characterized using SEM (see Figure 1C for 15 °C-SC and Figures S1–S4 in Supplementary Materials for the other samples). Their average diameters ranged from ca. 170 to 800 nm (see Table 1 and Figure S5 in Supplementary Materials) with a clear decreasing trend with the rising temperature of the synthesis as it has been also reported previously for similar systems [3,27,28]. This enables fine-tuning of the size of the particles according to needs. Moreover, the distributions of the sizes were relatively narrow, as the standard deviations of the average diameters did not exceed 7% for the samples, and as such the particles may be treated as monodisperse ones. 

**Figure 2:**
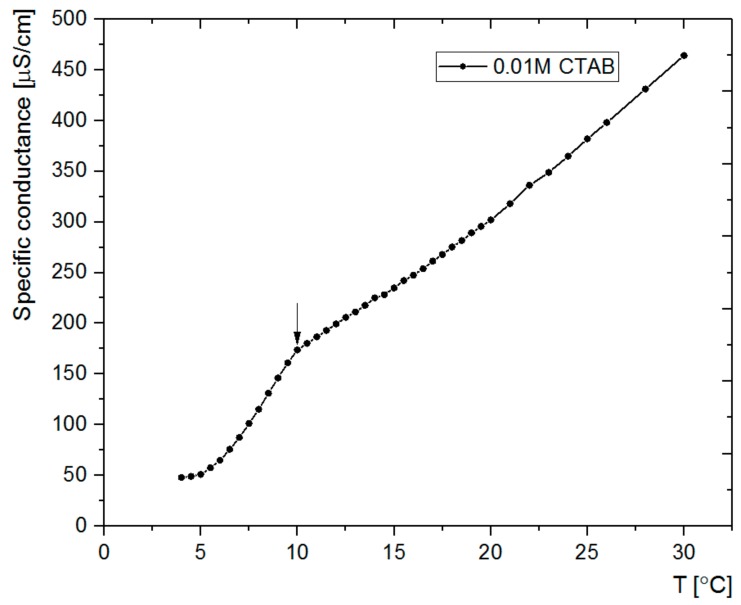
Specific conductivities of CTAB solutions (0.01 M) in the water/ethanol (25%) mixture measured at various temperatures. The arrow indicates the Krafft temperature.

A somewhat similar trend was observed regarding the dependence of the shell on the temperature. The thickness varied between 11 and 80 nm, saturating at a lower limit of 45 °C and a higher limit of 15 °C (see Table 1). Thus, the shell thickness may also be tuned in the studied temperature range, although the temperature dependence of the shell thickness does not seem to be as clear as for the growth of the solid cores. It should be related to the dependence of critical micelle concentration (CMC) on the temperature. For ionic surfactants, CMC decreases with increasing temperature, reaching a minimum value and then increasing [25]. It should be also dependent on the T_K_ that for CTAB in an aqueous solution was reported to be between 20 and 25 °C [29]. This temperature should be lower for the water/ethanol mixture applied in the current study. The surfactant concentration (0.01 M) used here was only ca. five times higher than the CMC of CTAB in the water/ethanol (ca. 25% of ethanol) mixture (ca. 0.002 M) [30]. Conductivity measurements, as a function of temperature, were performed for CTAB solutions in water/ethanol (25% ethanol) in order to show how T_K_ depends on the concentration of CTAB in the studied mixture of solvents. The results showed that T_K,_ taken as the temperature at which a sharp change in the conductivity versus temperature plot occurred, is equal to 10 °C in the applied solvent (see Figure 2). These observations correlate with the occurrence of the precipitate at the mentioned temperatures, thus confirming that T_K_ was indeed determined. In fact, for the sample obtained at the temperature (8 °C) slightly below the determined T_K_, the mesoporous shell was found to be thinner (ca. 74 nm) than the one obtained at 15 °C (ca. 80 nm). It should be related to the lower concentration of the surfactant at 8 °C disabling the formation of micelles, as it was similarly reported for non-ionic surfactants [31].

**Figure 3:**
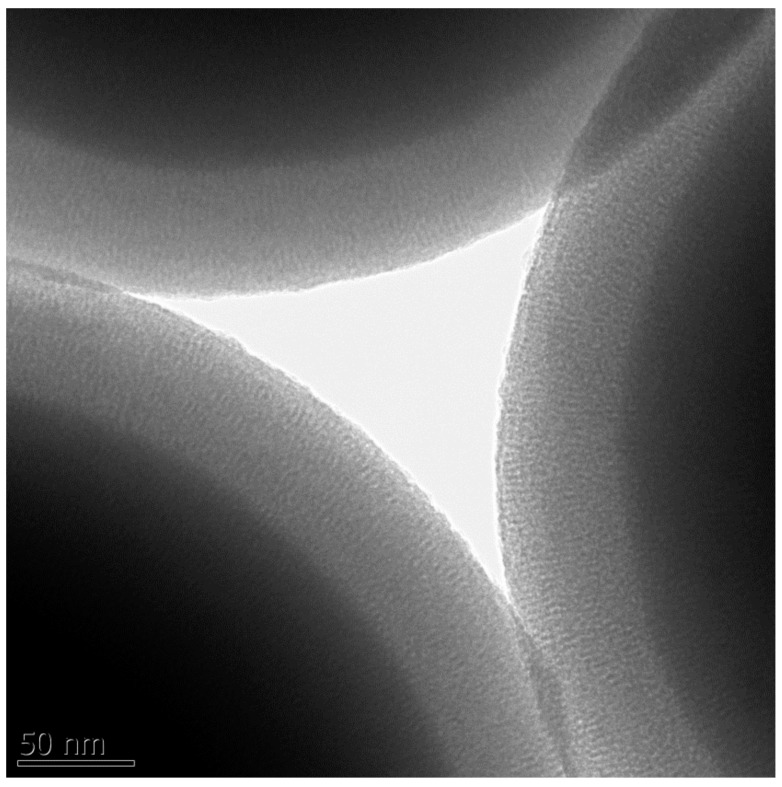
TEM image of 22 °C-SCMS particles.

Importantly, the pores in the shell were found to be well ordered in the radial direction (Figure 3). Moreover, the shell thickness was found not to depend on the reaction time (above 6 h), as it was checked for the samples grown at 22 °C by varying the time of the shell growth from 6 to 48 h. It indicates that the ordering of the micellar templates leading to the shell of a given thickness is governed in the studied system mainly by the applied temperature. Thus, the desired core and shell sizes may be easily tuned by setting the temperature of both synthetic steps following the trend line presented in Figure S5. 

### 3.2. FT-IR Spectral Analysis

**Figure 4:**
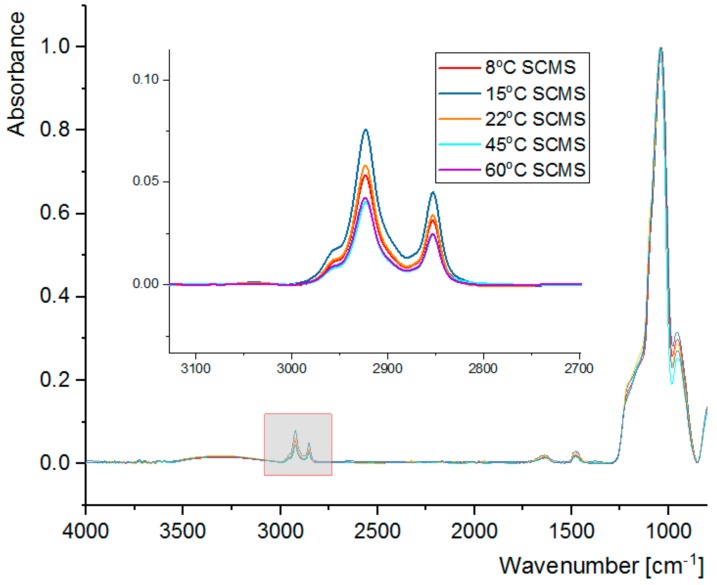
FT-IR spectra for SCMS particles synthesized at various temperatures.

The synthesized SCMS particles were subjected to FT-IR analysis (Figure 4). The spectra show, among others, very intense bands at 1040 cm^−1^, characteristic of Si-O vibrations, and at 2853 cm^−1^ and 2923 cm^−1^, which can be assigned to the C-H vibrations of the template surfactant molecules. Absorbance of the band at 1040 cm^−1^ was normalized for all SCMS samples, thus enabling quantitative comparison of the absorbance of bands assigned to the surfactant molecules. It can be observed that the amount of the CTAB surfactant occluded in the SCMS particles depends on the temperature of the synthesis and is the highest at 15 °C. It decreases with the increase of temperature up to 45 °C and is smaller also for the 8 °C-SCMS. This relation correlates well with the dependence of the shell thickness on the temperature of the synthesis and can be similarly explained (see Table 1 and the discussion in Section 3.1.). Below T_K,_ the amount of the occluded surfactant is limited by solubility, but at higher temperatures less dense micellar structures seem to be formed involving less surfactant molecules per unit mass of silica as well. Such an observation may be associated with the reported increase of CMC with increasing temperature [30]. It is mainly related to the increasing repulsive interaction between the charged surfactant heads due to the increasing degree of counterion dissociation in the studied solvent mixture. The contribution of the decrease of microviscosity of the micelles with increasing temperature can also be taken into account [32]. 

The calcined particles (SCMS-C) were also subjected to FT-IR analysis in order to confirm successful removal of the organic template during calcination. A representative spectrum of 22 °C-SCMS-C, showing no C-H vibrations of the surfactant molecules, is presented in Figure S7. 

### 3.3. Nitrogen Adsorption Analysis

**Figure 5:**
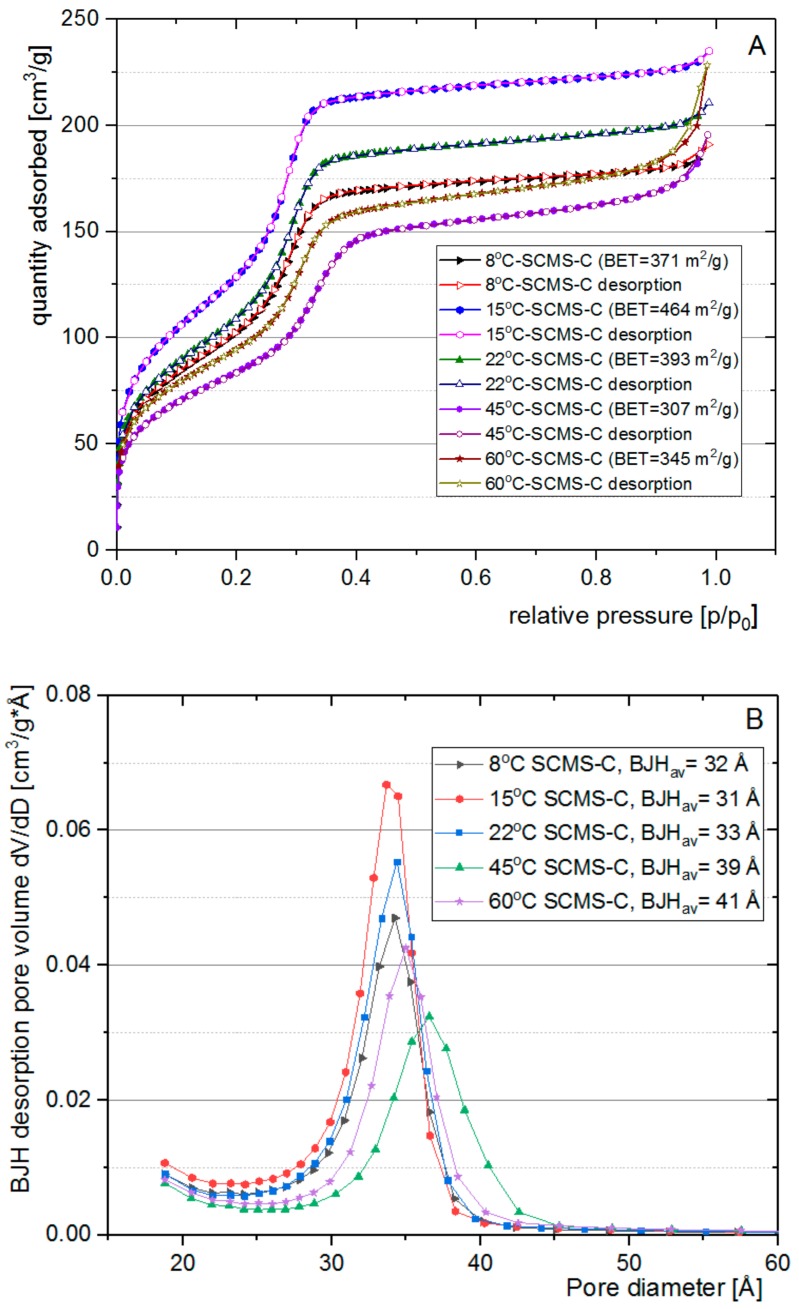
(**A**) N_2_ adsorption-desorption isotherms at 77 K for SCMS-C. (**B**) BJH (based on Barrett-Joyner-Halenda theory) pore distribution of SCMS-C particles.

Structural characterization was performed using nitrogen sorption for calcined core-shell samples (SCMS-C). Importantly, even after prolonged heating at 550 °C the particles retained their spherical shape (see Figure 1C and Figures S1–S4C), indicating the mechanical robustness of the formed structures. The isotherms for all the samples (Figure 5A) were found to be of the IVb IUPAC type [33] that is characteristic of mesoporous materials with pore sizes below approximately 4 nm. A small hysteresis loop in near-atmospheric pressure was observed only for the sample synthesized at the highest temperature (60 °C-SCMS-C). This hardly noticeable effect can be explained by the larger contribution of interparticle voids (i.e., the condensation step at high relative pressure) as compared to the other samples (see further). The lack of hysteresis loop may also indicate denser packing of the pores in the shells, especially at a low temperature synthetic condition, as opposed to the reported formation of large intergranular mesopores during the synthesis of particles even at room temperature [3]. 

The highest BET surface area, 464 m^2^/g, was found for the particles synthesized at 15 °C. The specific surface area of dense silica particles obtained using the sol-gel method is typically on the level of a few m^2^/g for the particles of large sizes like studied here [34]. Thus, we could neglect the contribution of the cores in the surface area of SCMS particles. The sample prepared at 8 °C, the temperature lower than T_K_, showed ca. 25% lower surface area, indicating that further cooling of the reaction mixture reduces the ability of a porous structure to form, likely due to the limiting solubility of the surfactant at such temperature. However, with the increasing temperature of the synthesis above 15 °C the surface area of the formed particles also decreases down to 307 m^2^/g for 45 °C-SCMS-C and 345 m^2^/g for 60 °C-SCMS-C. Such an observation may be associated with the reported increase of CMC with increasing temperature as explained in Section 3.2. Thus, for maximizing the surface area, the temperature of the synthesis of SCMS particles should be as low as possible but above T_K_.

**Figure 6:**
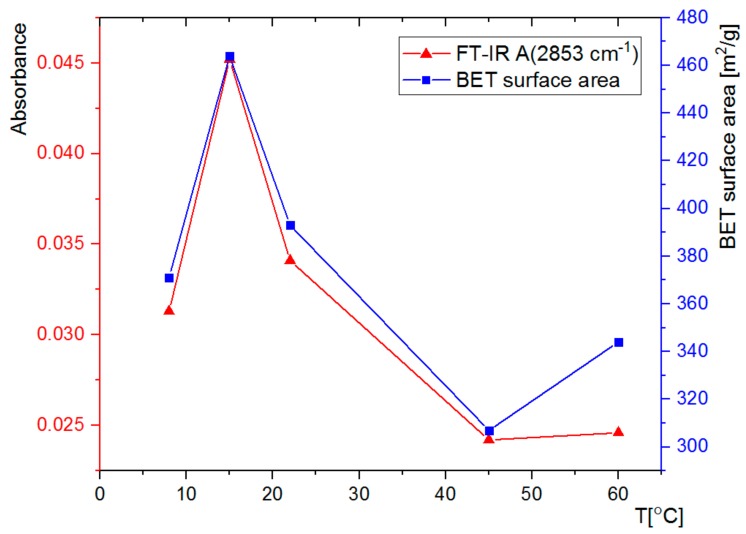
Dependences of absorption of the 2853 cm^−1^ band from the FT-IR spectra of the SCMS and BET (based on Brunauer-Emmett-Teller theory) surface area of SCMS-C particles synthesized at various temperatures.

This conclusion may be further supported by the dependence of the pore size distribution on the temperature of the synthesis (Figure 5B). The distributions, calculated using the BJH model, were found to be narrow with average pore sizes in the 3–4 nm range, typical for such mesoporous materials [23,24,35]. While lower synthetic temperatures lead to average pore size oscillations around 3.2 nm, for the shells synthesized at 45 and 60 °C the average size increased slightly to ca. 4.0 nm. The highest average pore size observed for 60 °C-SCMS-C should be partially caused by the formation of interparticle voids that were observed only for this sample (see Figure S6 in Supplementary Materials, as the pore size distribution in the mesopores region is comparable with the other samples. Such observations can be explained by the formation of less ordered micellar templates at higher synthetic temperatures. It may be further supported by comparing the amount of surfactant (based on FT-IR spectra) with BET surface area, which shows a strong correlation (Figure 6). 

Although hexagonal MCM-41 type silica particles, synthesized without cores, may exhibit higher BET-surface area than SCMS particles, the values reported here are relatively high, taking into account the contribution of nonporous solid cores. Moreover, the mechanical robustness provided by the presence of the cores in SCMS is very important for their potential applications as submicrometer reactors or recyclable carriers of, e.g., catalysts. The SCMS particles could be easily and repeatedly isolated from the aqueous suspensions, at moderate forces (centrifugation speed), and short times that are typically not sufficient for less dense particles. Moreover, large forces might be destructive for mesoporous phases and/or lead to the irreversible aggregation of particles that is avoided in SCMS material. 

### 3.4. Adsorption of Rh6G within SCMS Particles

**Figure 7:**
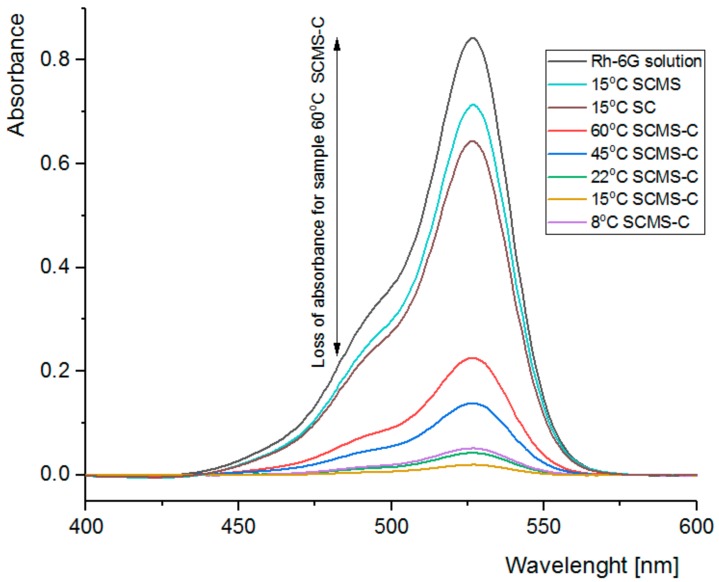
UV-VIS spectra of the starting Rh6G solution and respective solutions after adsorption of the dye in suspensions of various particles.

**Figure 8:**
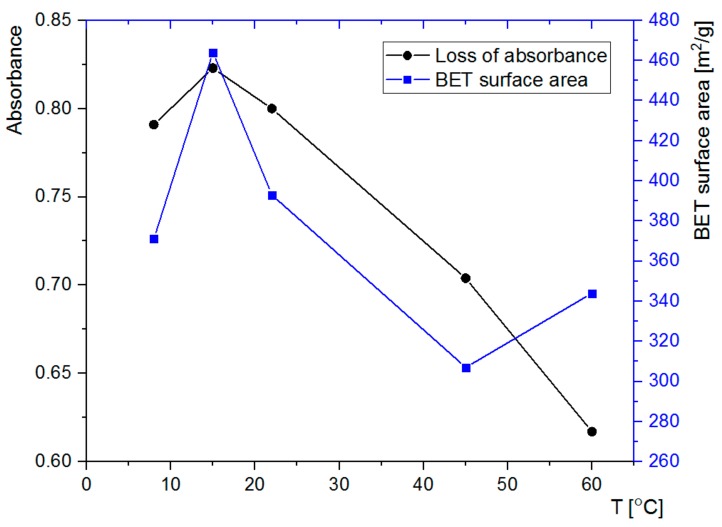
(Left axis) loss of absorbance (at ca. 527 nm based on the absorption spectra in Figure 7) of the Rh6G solution after adsorption of the dye in suspensions of SCMS-C particles, synthesized at various temperatures and BET surface areas (right axis) for the same particles.

The ability of the formed SCMS-C particles to adsorb model compounds that may be further used as reagents or photosensitizers was tested using Rh6G. Rh6G is a highly fluorescent cationic dye, which absorbs visible light at ca. 450–550 nm and is able to adsorb on the negatively charged surfaces of the silica-based particles. The particles were immersed in the solution of Rh6G and decolorization of the supernatant, being the result of adsorption of the dye within the particles, was followed using UV/Vis absorption spectroscopy. Significant amounts of Rh6G were adsorbed within all the studied SCMS-C particles after 1 h of shaking (Figure 7). However, there are clear differences in the amount of adsorbed Rh6G that reached more than 95% for 15 °C-SCMS-C and only 73% for 60 °C-SCMS-C. Thus, the adsorption capability of the SCMS-C particles could be also correlated with other properties of the particles (e.g., BET surface area) that were found to depend on the temperature of the synthesis, with the exception of 60 °C-SCMS-C (Figure 8). It is possible that the particles synthesized at the highest temperature are composed of less ordered and more irregular pores with, e.g., varying diameter along their lengths. Such pores would be less accessible for large cationic dye molecules (its largest dimension is equal to 1.6 nm) [36], which electrostatically interact with the pores, than for much smaller nitrogen molecules, used in the sorption experiment for the determination of BET surface area. A dye molecule once adsorbed within the pore could limit transport and further adsorption of the large dye molecules, especially if the pore’s diameter varies along its length [37].

Importantly, SC particles without a mesoporous shell, or SCMS particles with the pores occupied by the surfactant molecules, were shown to adsorb significantly less than the calcined samples (see absorbance for 15 °C-SC and 15 °C-SCMS in Figure 7). Thus, the majority of the adsorbed dye must have been located inside the pores. 

The sorption ability is nicely correlated with the BET surface area of the samples (Figure 8) with the exception of 60 °C-SCMS-C. It is likely related to the formation of some macropores, noticed only for this sample, that seem to be too large to effectively keep the dye molecules adsorbed.

## 4. Conclusions

Monodisperse solid core mesoporous shell all silica particles with radially ordered pores were synthesized using a one-pot surfactant templated method at various temperatures. By varying the temperature in the 8–60 °C range, the diameter of the dense cores and thickness of the mesoporous shells could be easily tuned. The SCMS particles underwent calcination for the removal of cationic surfactant, leaving the mesopores with the average size of 3–4 nm. The BET surface area was also found to significantly depend on the temperature of synthesis, reaching 464 m^2^/g for 15 °C. It was shown that lowering the temperature below the Krafft point (10 °C), determined for the used CTAB surfactant in the applied solvent mixture, lead to the formation of particles with lower content of surfactant, implying also a smaller specific surface area and weaker sorption abilities as tested using the model cationic dye (rhodamine 6G). However, for temperatures higher than 15 °C the mentioned properties of the particles were also smaller due to lower density or increasing destabilization (larger CMC for higher temperatures) of the micellar templates. Thus, although synthesis of the solid silica cores may be performed using this method at various temperatures, tailoring its diameter (ca. 170–800 nm) for a given application, the best mesoporous shells, in terms of sorption abilities, should be prepared at low temperatures, slightly above the Krafft temperature. The presented low temperature strategy may be extended for the synthesis of other core-shell systems that can be tailored for specific applications such as submicrometer carriers or reactors that require not only high specific surface area but also mechanical robustness for, e.g., facile isolation from suspensions. Moreover, by varying the solvent composition, one may further tune the solubility of a surfactant (Krafft temperature) and proceed with syntheses at even lower temperatures that may result in thicker and better ordered mesoporous shells. 

## Figures and Tables

**Table 1:** Sizes of the SCMS particles and estimated shell thickness for various temperatures of their synthesis as determined from SEM images.

Temp [°C]	d_sc_ [nm]	d_scms-c_ [nm]	Shell Thickness ^a^ [nm]
8	793 ± 30	941 ± 27	74
15	614 ± 40	773 ± 33	80
22	451 ± 23	531 ± 30	40
45	236 ± 13	257 ± 15	11
60	173 ± 10	194 ± 11	11

^a^ It was determined as a half of the difference between the average diameters of the respective SCMS-C and SC particles.

## Supplementary Materials

The following are available online at http://www.mdpi.com/2079-4991/8/4/230/s1, Figure S1: Distributions of the diameters (A) determined from respective SEM images and representative SEM images (B) for SC and (C) for SCMS-C synthesized at 8 °C, Figure S2: Distributions of the diameters (A) determined from respective SEM images and representative SEM images (B) for SC and (C) for SCMS-C synthesized at 22 °C, Figure S3: Distributions of the diameters (A) determined from respective SEM images and representative SEM images (B) for SC and (C) for SCMS-C synthesized at 45 °C, Figure S4: Distributions of the diameters (A) determined from respective SEM images and representative SEM images (B) for SC and (C) for SCMS-C synthesized at 60 °C, Figure S5: The dependence of the diameters of SC and SCMS-C particles on the temperature of their synthesis, Figure S6: BJH pore distributions of 45 °C-SCMS-C and 60 °C-SCMS-C nanoparticles, Figure S7: FT-IR spectrum of 22 °C-SCMS-C.
