# Supplementary material for: Tailored Synthesis of Core-Shell Mesoporous Silica Particles—Optimization of Dye Sorption Properties
Source: Nanomaterials (Basel). 2018 Apr 10;8(4):230. doi: 10.3390/nano8040230 (PMC5923560; doi:10.3390/nano8040230)
Supplement: Supplementary file 1 [file nanomaterials-08-00230-s001.pdf]

Supplementary Information

Tailored synthesis of core-shell mesoporous silica particles - optimization of dye sorption properties

Andrzej Baliś, Szczepan Zapotoczny

Jagiellonian University, Faculty of Chemistry, Gronostajowa 2, 30-387 Krakow, Poland

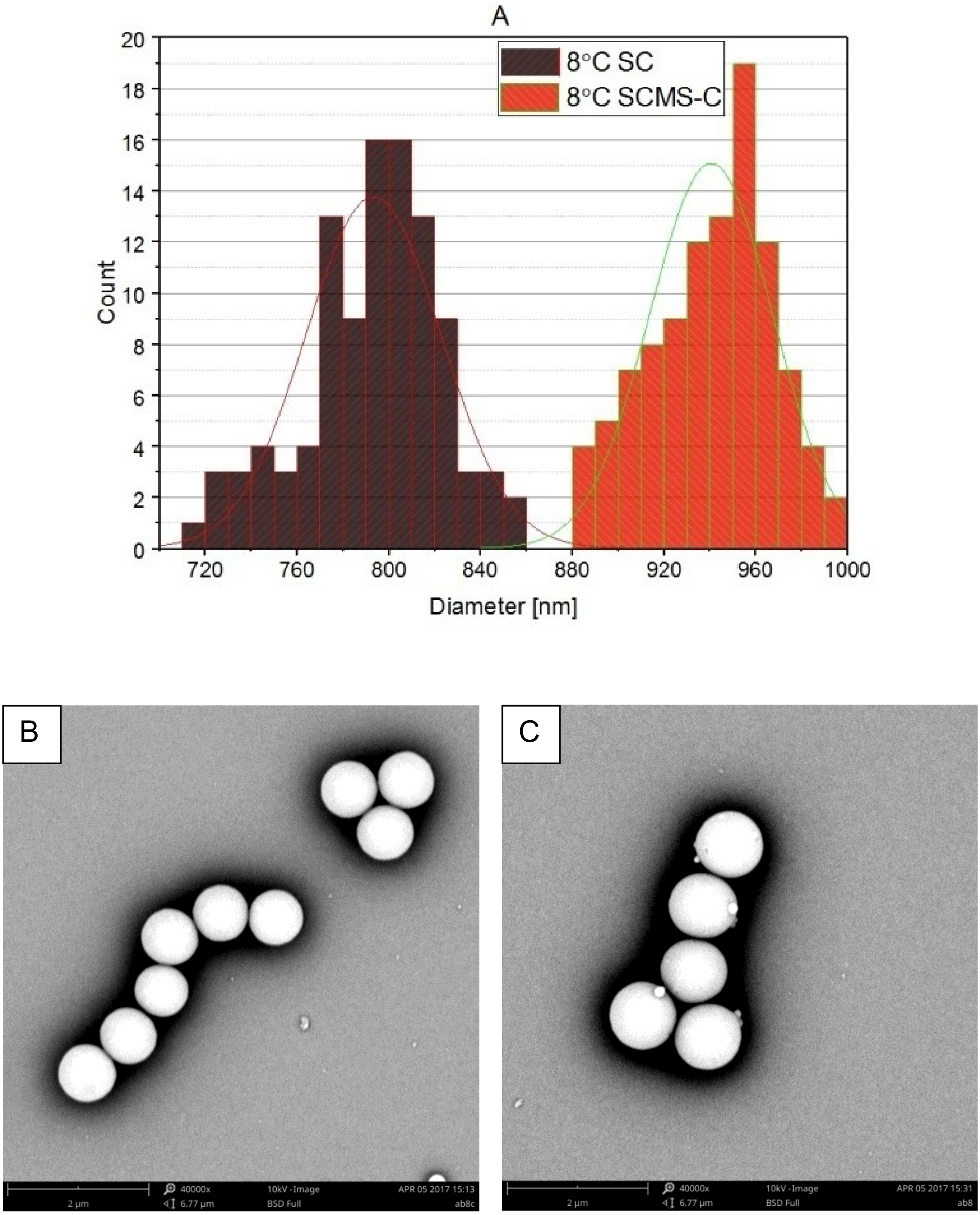

Figure S1. Distributions of the diameters (A) determined from respective SEM images and representative SEM images (B) for SC and (C) for SCMS-C synthesized at 8°C.

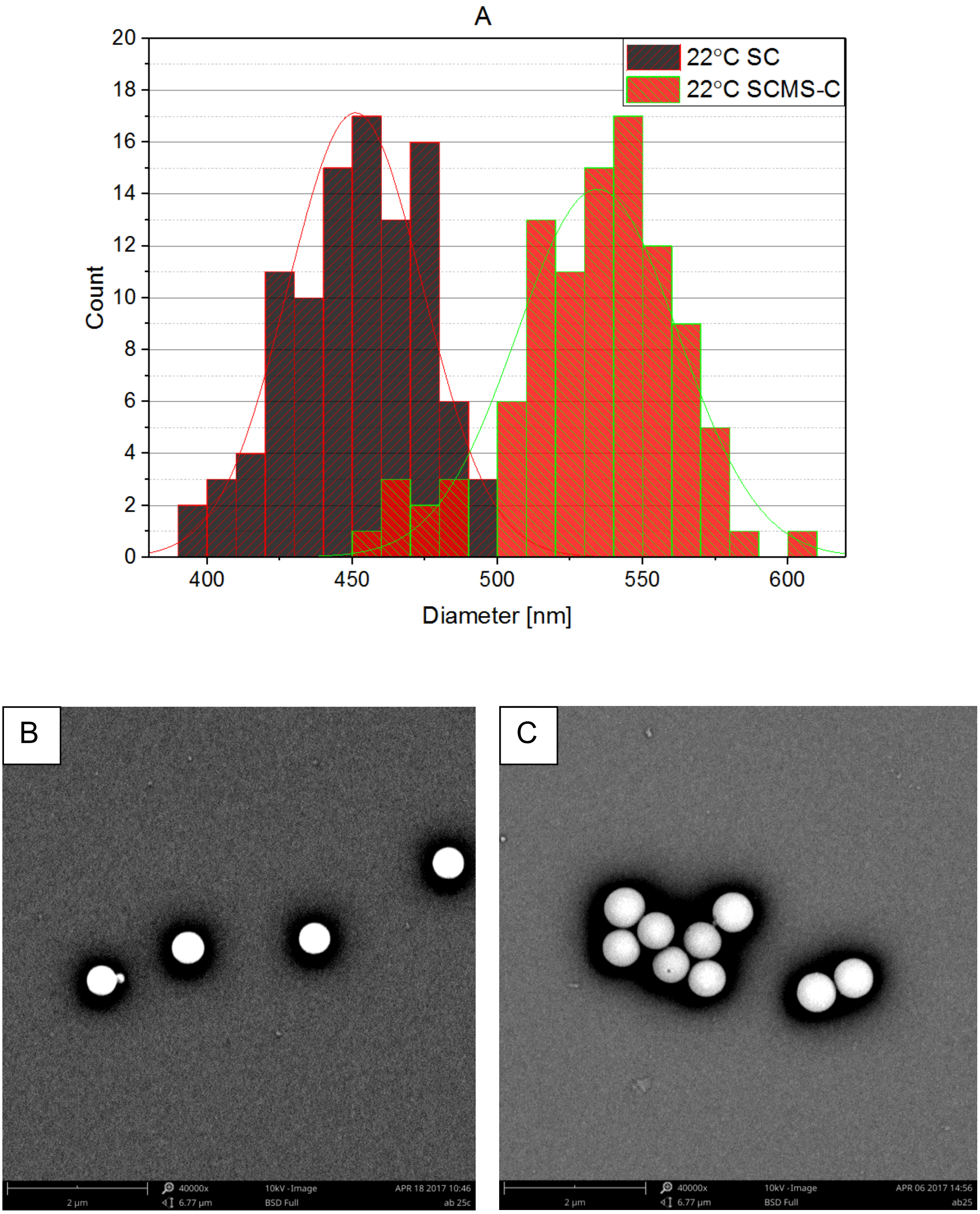

Figure S2. Distributions of the diameters (A) determined from respective SEM images and representative SEM images (B) for SC and (C) for SCMS-C synthesized at 22°C.

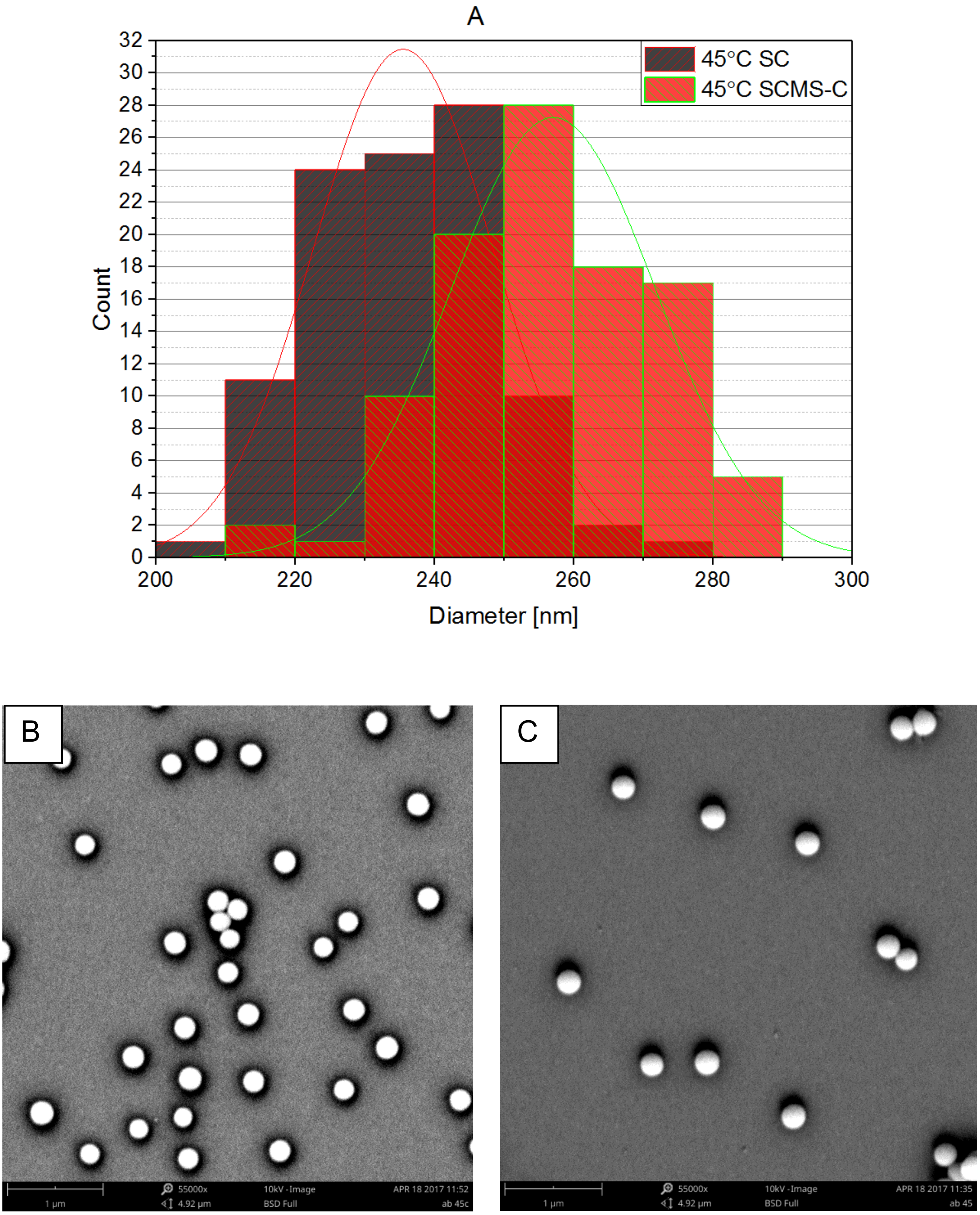

Figure S3. Distributions of the diameters (A) determined from respective SEM images and representative SEM images (B) for SC and (C) for SCMS-C synthesized at 45°C.

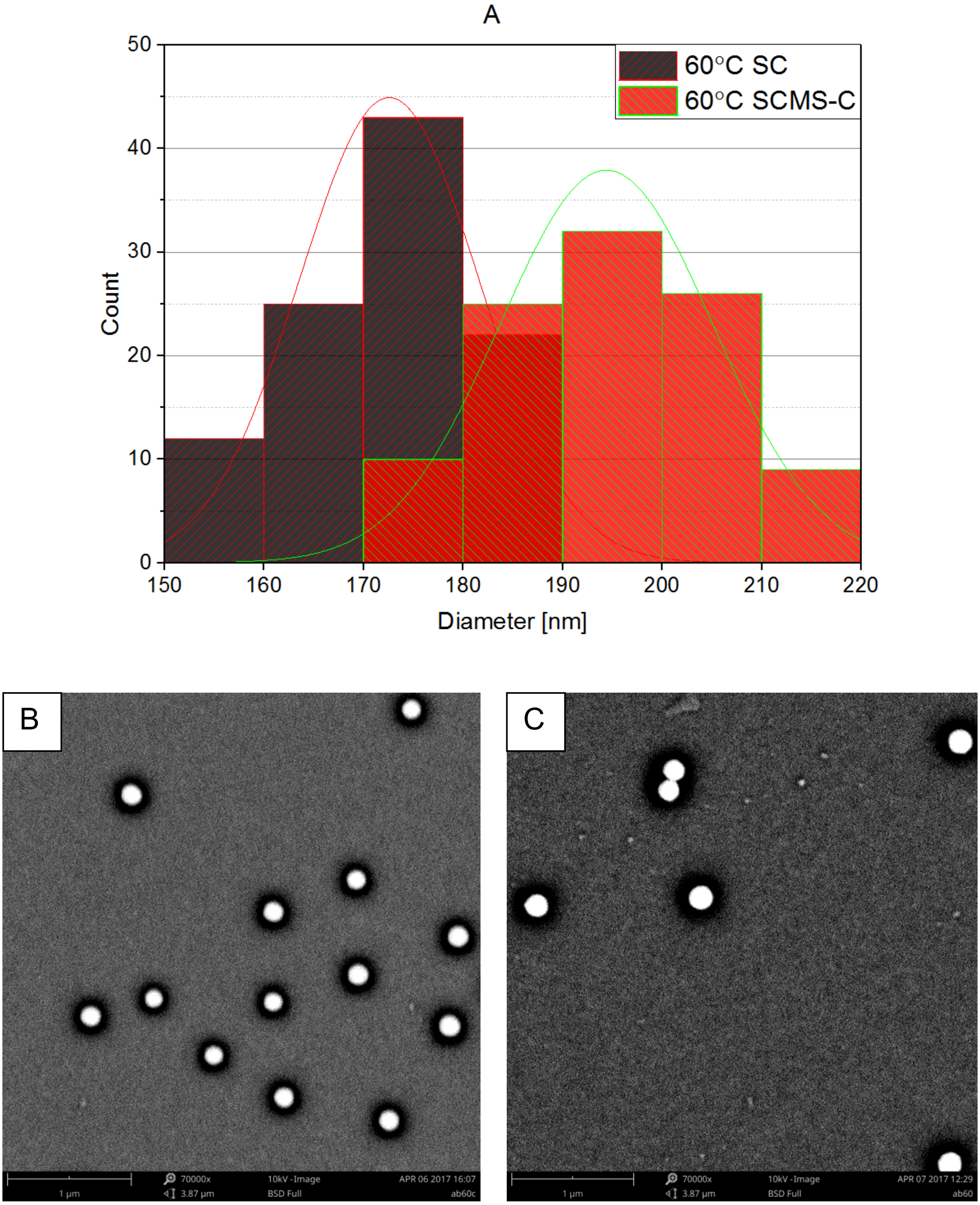

Figure S4. Distributions of the diameters (A) determined from respective SEM images and representative SEM images (B) for SC and (C) for SCMS-C synthesized at 60°C.

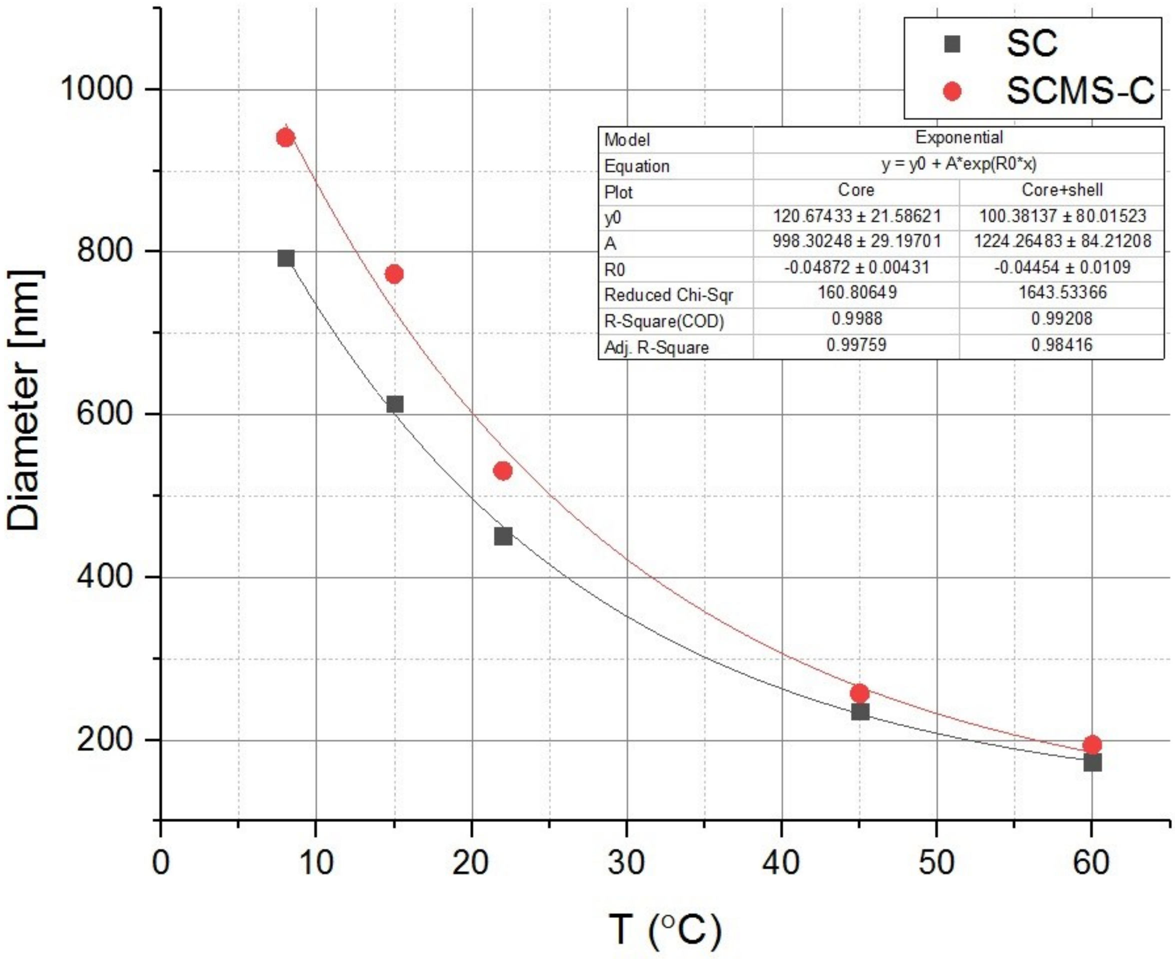

Figure S5. The dependence of the diameters of SC and SCMS-C particles on the temperature of their synthesis.

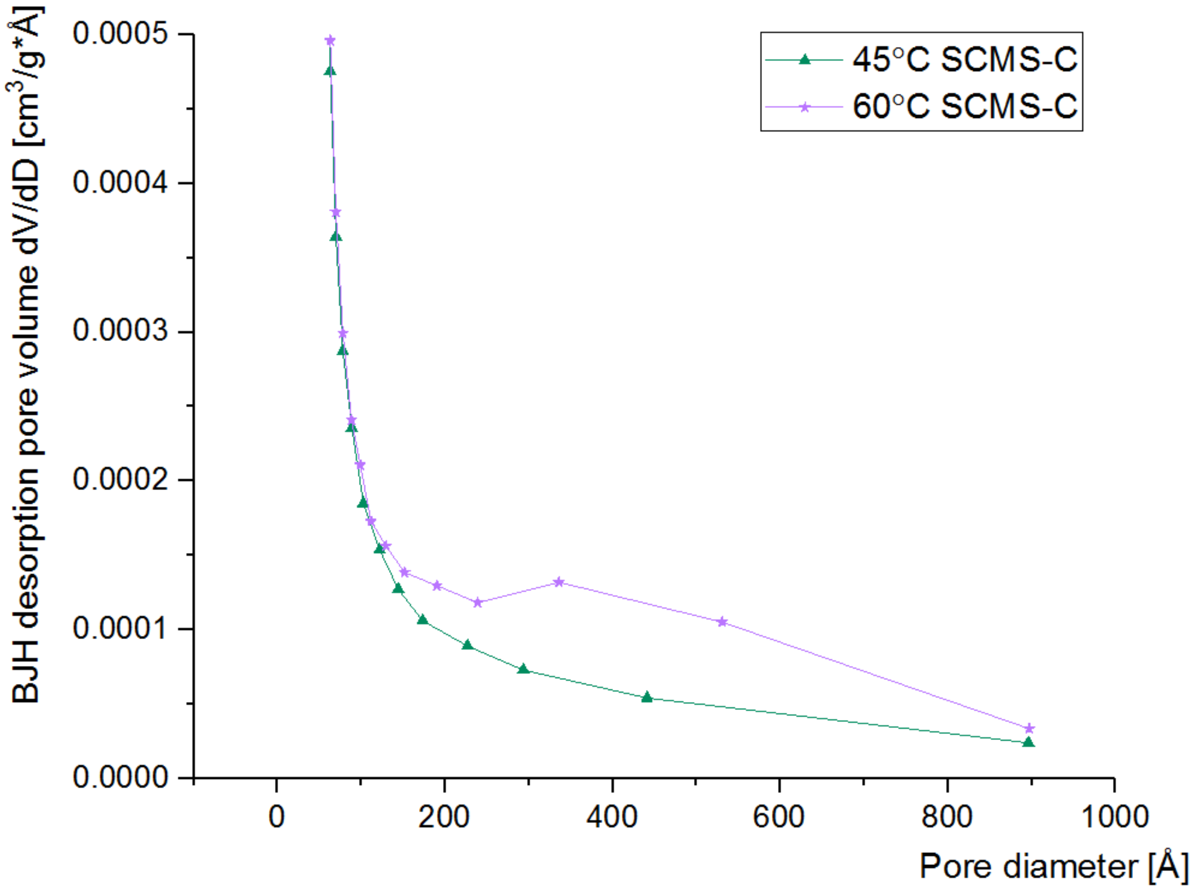

Figure S6. BJH pore distributions of 45°C-SCMS-C and 60°C-SCMS-C nanoparticles.

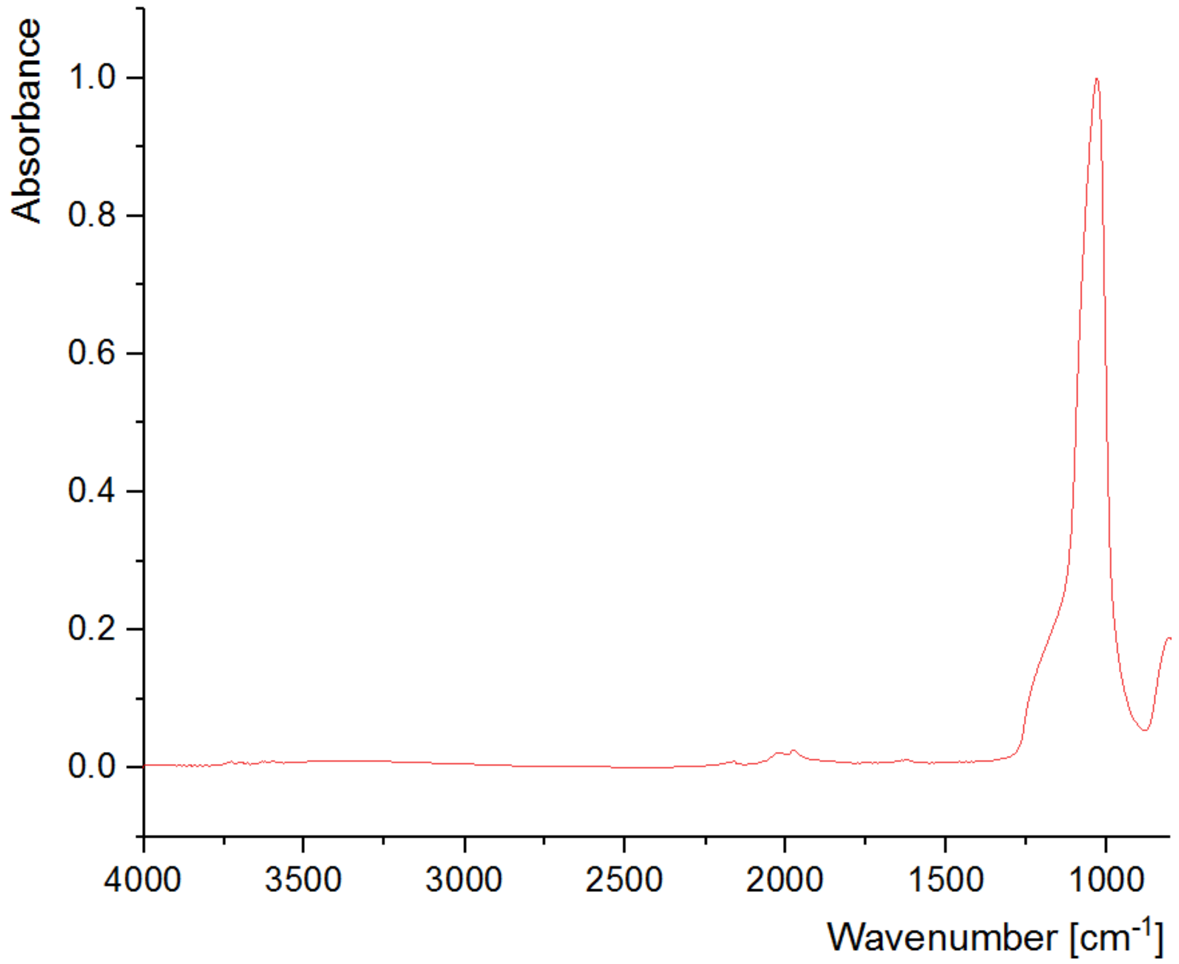

Figure S7. FT-IR spectrum of 22°C-SCMS-C.
